# Associations of long non-coding RNAs HOTAIR, LINC00951, POLR2E and HULC polymorphisms with the risk of esophageal and esophagogastric junction cancer in a western population: a case-control study

**DOI:** 10.1007/s11033-024-09206-0

**Published:** 2024-02-01

**Authors:** Efstratia Baili, Maria Gazouli, Andreas C. Lazaris, Prodromos Kanavidis, Maria Boura, Adamantios Michalinos, Alexandros Charalabopoulos, Theodore Liakakos, Andreas Alexandrou

**Affiliations:** 1 Upper Gastrointestinal and General Surgery Unit, First Department of Surgery, Laiko General Hospital, School of Medicine, National and Kapodistrian University of Athens, Agiou Thoma 17, Athens, 11527 Greece; 2 King’s Health Partners, London, UK; 3 Laboratory of Biology, School of Medicine, National and Kapodistrian University of Athens, Athens, Greece; 4 First Department of Pathology, School of Medicine, National and Kapodistrian University of Athens, Athens, Greece; 5 Department of General Surgery/Anatomy, School of Medicine, European University of Cyprus, Nicosia, Cyprus

**Keywords:** Single-nucleotide-polymorphisms (SNPs), Esophageal cancer, Esophagogastric junction carcinoma, Long noncoding RNAs (lncRNAs), HOTAIR rs920778, LINC00951 rs11752942, POLR2E rs3787016 and HULC rs7763881

## Abstract

**Background:**

The incidence of single-nucleotide-polymorphisms with malignant potential in esophageal cancer tissues has only been sparsely investigated in the west. Hence, we explored the contribution of four long non-coding RNAs’ polymorphisms HOTAIR rs920778, LINC00951 rs11752942, POLR2E rs3787016 and HULC rs7763881 in esophageal cancer susceptibility.

**Methods and results:**

Formalin-fixed paraffin-embedded tissue specimens from 95 consecutive patients operated for esophageal/esophagogastric junction carcinoma during 25/03/2014-25/09/2018 were processed. Demographic data, histopathological parameters, surgical and oncological outcomes were collected. DNA findings of the abovementioned population were compared with 121 healthy community controls. Both populations were of European/Greek ancestry. Sixty-seven patients underwent Ivor Lewis/McKeown esophagectomy for either squamous cell esophageal carcinoma (*N* = 6) or esophageal/esophagogastric junction Siewert I or II adenocarcinoma (*N* = 61). Twenty-eight patients were subjected to extended total gastrectomy for esophagogastric junction Siewert III adenocarcinoma. Neither LINC00951 rs11752942 nor HULC rs7763881 polymorphisms were detected more frequently in esophageal cancer patients compared with healthy community subjects. A significantly higher presence of HOTAIR rs920778 TT genotype in esophagogastric junction Siewert I/II adenocarcinoma was identified. POLR2E rs3787016 C allele and CC genotypes were overrepresented in the control group, and when found in esophageal cancer carriers were associated with earlier disease stages, as well as with minor lymph node involvement and lesser metastatic potential.

**Conclusions:**

HOTAIR rs920778 may serve as a potential therapeutic suppression target, while POLR2E rs3787016 may represent a valuable biomarker to evaluate esophageal cancer predisposition and predict treatment response and prognosis. Clinical implications of these findings need to be verified with further prospective studies with larger sample-size.

**Supplementary Information:**

The online version contains supplementary material available at 10.1007/s11033-024-09206-0.

## Introduction

Esophageal cancer (EC) is the seventh most common cancer worldwide with estimated 604,100 new cases in 2020 (3.1% of all sites), ranking sixth in overall mortality with 544,076 new deaths (5.5% of all sites), the latter signifying that in 2020 is estimated responsible for 1:18 cancer deaths as per GLOBOCAN Global Cancer Statistics 2021 [1].

EC global incidence as well as mortality rates are estimated two- to three-fold higher in men compared with women. This gender variation is followed by a striking geographic variation in both sexes. More than 15-fold differences between world regions exist, with rates ranging from 1.5:100,000 in Western Africa to 18.2:100,000 in Eastern Asia in men, and 0.4:100,000 in Central America to 6.8:100,000 in Eastern Asia in women [1]. This geographic incidence also differs substantially between the two most common histologic EC subtypes: esophageal squamous cell carcinoma (ESCC) and esophageal adenocarcinoma (EAC) which vary significantly in terms of etiology too. For ESCC, the so-far established main risk-factors are tobacco and alcohol consumption while additional suspected risk-factors are low socioeconomic status with poor diet quality of low fruit/vegetable intake, severe vitamins and micronutrients deficiencies, high polycyclic aromatic hydrocarbons (PAHs) consumption [2], betel quid chewing on the Indian subcontinent and hot mate drinking in Southern America [3]. Gastroesophageal reflux disease (GERD), Barrett’s esophagus, excess body mass index (BMI) and tobacco have been verified as key risk-factors for EAC [4].

While ESCC is broadly in decline, EAC incidence rates are rising rapidly [5]. This concerning EAC epidemic rise warrants further investigation as this cannot be exclusively attributed to the prevalence of the well-known EAC risk-factors, as obesity, GERD and tobacco. Although these environmental factors are deemed strongly implicated in EAC aetiopathogenesis, only a proportion of exposed individuals develop EAC, suggesting that genetic footprint may also contribute to malignant transformation of esophageal epithelial cells, so its role needs therefore to be reevaluated [6, 7].

Additional challenges health organizations encounter is that despite the milestones achieved in diagnosis and multimodal treatment, EC has still poor overall prognosis, with 5-year survival rates between 15–25% [8]. Delayed diagnosis to advanced stages and disease resistance to chemotherapy may contribute to these poor survival outcomes [9, 10]. Hence, an imperative need urges to identify novel molecular agents that could facilitate the promotion of EC cellular sensitivity to chemotherapy or immunotherapy regimens, as well as biomarkers that would expedite earlier cancer detection [11].

Aberrant expression of long non-coding RNAs (lncRNAs) is now well-established as associated with cancer development [12]. Emerging epidemiological evidence suggests that single-nucleotide-polymorphisms (SNPs) in certain genes influence the pathophysiology of human carcinogenesis including EC [13, 14]. Particularly, HOTAIR SNPs variations (as rs920778, rs4759314, rs1899663, rs12826786, rs874945, rs7958904 and rs10783618) have demonstrated a close connection to the development and progression of malignancies including EC and gastric cancer (GC), by acting as potential cancer susceptibility loci [15, 16]. However, although cumulative research is indicating possible relationship between HOTAIR SNPs and cancer risk, the so-far obtained results have been controversial, inconclusive, restricted to a specific ethnicity or limited by small sample-size. Others such as POLR2E rs3787016 and HULC rs7763881 polymorphisms have been associated with decreased risk of EC [17] in Asian populations.

The association of various SNPs in lncRNAs in EC tissues has only scarcely been investigated in the west. Based on previous research, we aimed to explore the potential contribution of four lncRNAs’ polymorphisms: HOTAIR rs920778, LINC00951 rs11752942, POLR2E rs3787016 and HULC rs7763881 in esophageal carcinoma susceptibility in a western population.

## Materials-methods

### Study design

This is a tertiary referral hospital-based case-control study designed according to the ‘Strengthening the Reporting of Observational Studies in Epidemiology’ guidelines for reporting observational studies (Table 1) [18]. A research protocol was developed and strictly followed by all participating authors/researchers. This was submitted to the Institutional Review Board of Laiko General Hospital and Ethics Committee of School of Medicine-National and Kapodistrian University of Athens (NKUA), Greece and approval was obtained prior to study start (IRB no: 18.01.2018/24). All procedures performed were in accordance with the ethical standards of the Helsinki Declaration 1964 and later versions. Informed consent was obtained from all patients prior to enrollment.

**Table 1:** STROBE Statement—Checklist of items that should be included in reports of case-control studies

	Item No	Recommendation	Page No
**Title and abstract**	1	(*a*) Indicate the study’s design with a commonly used term in the title or the abstract	1–3
		(*b*) Provide in the abstract an informative and balanced summary of what was done and what was found	3
*Introduction*			
Background/rationale	2	Explain the scientific background and rationale for the investigation being reported	4–5
Objectives	3	State specific objectives, including any prespecified hypotheses	5
*Methods*			
Study design	4	Present key elements of study design early in the paper	6
Setting	5	Describe the setting, locations, and relevant dates, including periods of recruitment, exposure, follow-up, and data collection	6
Participants	6	(*a*) Give the eligibility criteria, and the sources and methods of case ascertainment and control selection. Give the rationale for the choice of cases and controls	7
		(*b*) For matched studies, give matching criteria and the number of controls per case	n/a
Variables	7	Clearly define all outcomes, exposures, predictors, potential confounders, and effect modifiers. Give diagnostic criteria, if applicable	8–9
Data sources/measurement	8*	For each variable of interest, give sources of data and details of methods of assessment (measurement). Describe comparability of assessment methods if there is more than one group	8–9
Bias	9	Describe any efforts to address potential sources of bias	6–8, 18
Study size	10	Explain how the study size was arrived at	7–9
Quantitative variables	11	Explain how quantitative variables were handled in the analyses. If applicable, describe which groupings were chosen and why	8,10
Statistical methods	12	(*a*) Describe all statistical methods, including those used to control for confounding	10
		(*b*) Describe any methods used to examine subgroups and interactions	10
		(*c*) Explain how missing data were addressed	10
		(*d*) If applicable, explain how matching of cases and controls was addressed	10
		(*e*) Describe any sensitivity analyses	10
**Results**			
Participants	13*	(a) Report numbers of individuals at each stage of study—eg numbers potentially eligible, examined for eligibility, confirmed eligible, included in the study, completing follow-up, and analysed	10–11
		(b) Give reasons for non-participation at each stage	10–11
		(c) Consider use of a flow diagram	n/a
Descriptive data	14*	(a) Give characteristics of study participants (e.g. demographic, clinical, social) and information on exposures and potential confounders	10–11
		(b) Indicate number of participants with missing data for each variable of interest	10–11
Outcome data	15*	Report numbers in each exposure category, or summary measures of exposure	10–14
Main results	16	(a) Give unadjusted estimates and, if applicable, confounder-adjusted estimates and their precision (e.g., 95% confidence interval). Make clear which confounders were adjusted for and why they were included	10–14
		(b) Report category boundaries when continuous variables were categorized	10–14
		(c) If relevant, consider translating estimates of relative risk into absolute risk for a meaningful time period	n/a
Other analyses	17	Report other analyses done—eg analyses of subgroups and interactions, and sensitivity analyses	10–14
**Discussion**			
Key results	18	Summarise key results with reference to study objectives	15–18
Limitations	19	Discuss limitations of the study, taking into account sources of potential bias or imprecision. Discuss both direction and magnitude of any potential bias	18
Interpretation	20	Give a cautious overall interpretation of results considering objectives, limitations, multiplicity of analyses, results from similar studies, and other relevant evidence	18
Generalisability	21	Discuss the generalisability (external validity) of the study results	18
**Other information**			
Funding	22	Give the source of funding and the role of the funders for the present study and, if applicable, for the original study on which the present article is based	19

*Give information separately for cases and controls

**Note**: An Explanation and Elaboration article discusses each checklist item and gives methodological background and published examples of transparent reporting. The STROBE checklist is best used in conjunction with this article (freely available on the Web sites of PLoS Medicine at http://www.plosmedicine.org/, Annals of Internal Medicine at http://www.annals.org/, and Epidemiology at http://www.epidem.com/). Information on the STROBE Initiative is available at http://www.strobe-statement.org

The study was conducted over a nine-year period with the recruitment phase set between 25/03/2014-25/09/2018 and follow-up phase with end-date set at 30/06/2023. Rationale for determining the recruitment start-date was that, as of March 2014 the dedicated esophagogastric surgical team began operating at Laiko Hospital. Rationale for setting the end-date was that from October 2018 and onwards, we changed our practice from open to minimally invasive approach, so this was an effort to minimize bias that this shift in operating technique could potentially introduce to our oncological outcomes or subsequent survival analysis.

Two independent authors (EB, MB) extracted the data from our prospectively-collected Upper GastroIntestinal (UGI) Cancer database encompassing data from theatres, surgical/medical records, electronic/paper notes from inpatient and outpatient visits, investigations performed in public and private sectors. Discrepancies in data extraction were resolved after consensus with a third independent author (AM). All six surgeons participating in the study (EB, MB, AM, AC, TL and AA) were responsible to prospectively recruit, maintain accurate log and update patients’ records on our UGI Cancer database, as well as to conduct patients’ follow-up as per protocol.

Most senior molecular biologist (MG) designed and supervised the genotyping experiments. Most senior pathologist (ACL) supervised the histopathology examination and reports as well as the appropriate FFPEs tissue samples’ selection for the genotyping. Most senior surgeon (TL) supervised all surgical operations performed during the recruitment period.

### Patient selection: inclusion-exclusion Criteria

All consecutive adult patients who underwent surgery for histologically-confirmed malignancy involving the middle-third, lower-third part of the esophagus or the esophagogastric junction (EGJ) (Siewert I-III) [19] at the Department of UGI Surgery, Laiko General Hospital, School of Medicine-NKUA, Greece were deemed eligible for inclusion. Clinical and pathological staging, as well as all the definitions described in the present study follow the guidelines of the TNM staging system of the American Joint Committee on Cancer (AJCC), 8th edition [20]. All patients were risk-assessed and clinically staged with physical examination, computed tomography and gastroscopy. At the time of diagnosis, all were evaluated by the dedicated cancer Multidisciplinary Team at Laiko Hospital which formulated the appropriate multimodal treatment strategy as per international National Comprehensive Cancer Network (NCCN) [21] and European Society for Medical Oncology (ESMO) [22] guidelines.

Exclusion criteria were as follows: (a) non-adult subjects, (b) patients with cancer of the upper-third/cervical esophagus, (c) those submitted to emergency surgery, (d) esophagogastric malignancy family history.

Regarding our control group, community subjects were recruited from the Department of Molecular Biology, School of Medicine, NKUA, Greece. Cases and controls were unmatched; controls had no self-reported history of cancer at any site. Both were from European/Greek ancestry and resided in the geographical region of Greece.

### Data extraction: primary-secondary variables of interest

Primary study endpoint was to ascertain the presence of four lncRNAs’ polymorphisms: HOTAIR rs920778, LINC00951 rs11752942, POLR2E rs3787016 and HULC rs7763881 in primary esophageal and EGJ tumors in a western population, as Greece. We additionally sought to investigate the correlation of the aforementioned genetic values with the clinical, pathological, and oncological outcomes to identify further associations of these genetic footprints with recurrence patterns as well as metastatic potential in esophageal carcinogenesis process in a western population. Secondary endpoints were to assess the incidence of these SNPs in EAC and ESCC patients (subgroup analysis by histological subtype) and furthermore in EGJ Siewert I/II compared with EGJ Siewert III (subgroup analysis by tumor location).

To this end, variables of interest extracted from our UGI Cancer Database were: (1) demographics comprising age, gender, preoperative health of surgical candidates based on the American Society of Anesthesiologists’ classification of physical health grading system (ASA I-V) [23]; nutritional status and BMI according to the European Society for Clinical Nutrition and Metabolism (ESPEN) guidelines [24], (2) histology, size, location of primary tumor, neoadjuvant chemo-radiotherapy if offered; (3) date/type of surgical operation, lymphadenectomy extent, surgical time (minutes), estimated blood loss (EBL, ml), length of hospital stay (LOS, days); (4) final histopathological characteristics as tumor size, location and extent, lymph node (LN) harvest and infiltration, histological type, grade and stage, lymphovascular invasion, neoadjuvant treatment effect, resection (R1-3) and circumferential resection margins (CRM, mm), as per World Health Organization (WHO) and College of American Pathologists (CAP) recommendations [25]; (5) (%) minor/major complications (90-days), in-hospital mortality (90-days), follow-up length (months), adjuvant treatment where applicable, date/type of recurrence, disease-free survival (DFS, months) and overall survival (OS, months).

Minor complications were defined as Grade < II and major as Grade > IIIa based on the Clavien-Dindo severity classification system [26]. Recurrence date was set as the date of first investigation documenting the recurrence/metastasis. DFS was defined as the period from operation date and first recurrence date. OS was defined as the period between operation date and patient’s death.

### Sample collection and preparation for genetic analysis

Surgical tissue specimens for all enrolled patients were transferred after completion of surgical operation to the First Department of Pathology, School of Medicine, NKUA, Greece. After gross pathologic examination and marking of the margins, each specimen was formalin fixed and underwent tissue processing for paraffin embedding. Standard fixation methods to preserve nucleic acid integrity were used including 10% neutral-buffered formalin fixed for 24–72 h. Once paraffin embedded, the tissue samples were then sectioned with a microtome and placed on a glass slide to formulate microscopic slides ready to be viewed under the microscope by the pathologists. When necessary, the embedding process was reversed to get the paraffin wax out and allow for staining of the sections, as Hematoxylin and Eosin (H&E) staining. All tissue samples were reviewed by two independent pathologists. Most senior pathologist (ACL) examined all the microscopic slides for each specimen and selected the slide and its corresponding FFPE tissue block with the higher tumor burden in preparation for nucleic acid extraction.

### Genotyping of HOTAIR rs920778, LINC00951 rs11752942, POLR2E rs3787016 and HULC rs7763881

The nominated FFPE tissue blocks with the highest tumor burden from all recruited patients were transferred to Molecular Biology Laboratory, School of Medicine, NKUA, Greece. The percentage of tumor cells in each sample was minimum 50%. One to two − 1 mm diameter punches were sampled from the FFPE blocks. The punches were deparaffinized, homogenized and proteinase K digested. Then, the genomic DNA/RNA extraction was performed using a commercial RNA Extraction Kit from FFPE Samples (NucleoZOL, Macherey-Nagel, Germany). LncRNAs genotypes were identified through the “polymerase chain reaction-restriction fragment length polymorphism” (PCR-RFLP) or allele specific PCR depending on the SNP. Most senior molecular biologist (MG) supervised the experiments as per published methodology [27].

### Statistical analysis

Descriptive statistical analysis was conducted for all the encountered parameters, measuring the accumulated values. All variables are reported as means and medians with their corresponding standard deviations, ranges and proportions. We assessed the relationship between lncRNAs’ gene polymorphisms and EC, EAC-EGJ and ESCC cancer susceptibility by determining the genotype and allele frequencies of all cases and controls. Genotype frequencies were compared using the Fisher’s exact test with Yate’s continuity correction. Odds ratios (ORs) and 95% confidence intervals (95% CI) were calculated, using the approximation of Woolf. To summarize the ORs of the four polymorphisms we applied five genetic models: allele contrast, homozygous, heterogeneous, dominant and recessive models (AA, homozygotes for the common allele; AB, heterozygotes; BB, homozygotes for the rare allele). Correlations between SNPs and clinicopathological parameters were also statistically analyzed. Survival analysis was performed by using Kaplan-Meier curves and multivariate comparisons by using cox proportional hazards models. The probability p-values were two tailed and *p* < 0.05 was adopted as the statistically significant level. Censoring date was 30/06/2023. Statistical analysis was performed with R, version 4.0.4 (R Project for Statistical Computing).

## Results

### Study Population, Clinicopathological, Surgical and Oncological outcomes

All enrolled study subjects were adults of European/Greek ancestry divided into two groups incorporating FFPE tissue samples from *N* = 95 consecutive esophageal/EGJ cancer patients subjected to surgical treatment as a case-group and blood samples from *n* = 121 cancer-free community subjects as a control-group. The characteristics of the surgical-group are listed in Table 2.

**Table 2:** Demographics-Surgical/Oncological outcomes for the whole Esophageal Cancer (EC) cohort (*N* = 95 patients)

Variables	Value N (%)
Age (Mean ± SD, years) Median (range), years	62.9 ± 11.39 63 (27–83)
Gender: Male Female	Ν = 86 (90.5%) Ν = 09 (9.5%)
ASA Score: I II III IV	Ν = 31 (32.6%) Ν = 46 (48.4%) Ν = 15 (15.8%) Ν = 03 (3.2%)
Chemotherapy/Chemoradiotherapy: Neoadjuvant Adjuvant	*N* = 24 (25.3%) *N* = 55 (57.9%)
Tumor Location: MT Esophagus LT Esophagus EGJ-Siewert I EGJ-Siewert II EGJ-Siewert III	*N* = 02 (2.1%) *N* = 08 (8.4%) *N* = 20 (21.1%) *N* = 44 (46.3%) *N* = 21 (22.1%)
Operative technique: Open IL 2s-esophagectomy Open MK 3s-esophagectomy Total extended gastrectomy	*N* = 48 (50.5%) *N* = 19 (20%) *N* = 28 (29.5%)
Histological Type: Adenocarcinoma Adeno-squamous Squamous Cell Carcinoma MANEC High Grade Dysplasia	*N* = 84 (88.4%) *N* = 02 (2.1%) *N* = 06 (6.3%) *N* = 01 (1.1%) *N* = 02 (2.1%)
Tumor Differentiation: Poorly-differentiated (G3) Moderately-differentiated (G2) Well-differentiated (G1) Cannot be assessed (Gx) or N/A	*N* = 52 (54.7%) *N* = 36 (37.9%) *N* = 02 (2.1%) *N* = 05 (5.3%)
Final pathological TNM staging: 0 I II III IV	*N* = 04 (4.2%) Ν = 10 (10.5%) Ν = 18 (19%) Ν = 42 (44.2%) *N* = 21 (22.1%)
Tumor (T) status: pT0 pTis pT1 pT2 pT3 pT4	*N* = 01 (1.1%) Ν = 03 (3.2%) *N* = 07 (7.4%) *N* = 19 (20%) *N* = 54 (56.8%) *N* = 11 (11.5%)
Lymph Node (N) status: N0 N1 N2 N3	Ν = 29 (30.5%) Ν = 13 (13.7%) Ν = 22 (23.2%) Ν = 31 (32.6%)
Lymph node harvest: >15 <15	*N* = 87 (91.6%) *N* = 08 (8.4%)
Resection Status: R0 R1 R2	Ν = 86 (90.5%) Ν = 09 (9.5%) Ν = 00 (0%)
Circumferential Resection Margin (CRM): Negative Positive	Ν = 85 (89.5%) Ν = 10 (10.5%)
Clavien-Dindo Complications (90-day): None I II IIIa IIIb IVa IVb V	Ν = 48 (50.5%) *N* = 04 (4.2%) *N* = 19 (20%) *N* = 14 (14.7%) *N* = 01 (1.1%) *N* = 02 (2.1%) *N* = 00 (0%) *N* = 07 (7.4%)
Type of 1st disease progression: Local recurrence Regional LN metastasis Distant Metastasis Combined	*N* = 02 (2.1%) *N* = 10 (10.5%) *N* = 32 (33.7%) *N* = 05 (5.3%)
Median Disease Free Survival (months, range)	18.4 (2–97)
Median Overall Survival (months, range)	32.5 (4–97)
Median Length of Follow-up (months, range )	36 (4–97)

Notes: MT: Middle Thoracic Esophagus, LT: Lower Thoracic Esophagus, EsophagoGastric Junction (EGJ), 2s: two stage, 3s: three stage, IL: Ivor Lewis (laparotomic/thoracotomic), MK: McKeown (laparotomic/thoracotomic/left neck), MANEC: Mixed adeno-neuroendocrine carcinoma, N/A: Not Applicable

Mean age of the patients at time of surgery was 62.9 years with median ASA Class II (range I-IV), most were male (Ν = 86/95, 90.5%). Primary tumor location was at middle-thoracic esophagus, lower-thoracic esophagus, EGJ-Siewert I, EGJ-Siewert II and EGJ-Siewert III in 2/95, 8/95, 20/95, 44/95 and 21/95 patients respectively. As per AJCC-8th Edition, cancers crossing the EGJ with their epicenter in the proximal 2 cm of the stomach (EGJ-Siewert II) were staged and treated as EC, whereas cancers crossing the EGJ with their epicenter in the proximal 2 to 5 cm of the stomach (EGJ-Siewert III) were staged and treated as GC. As such, surgical operations performed were: Ivor Lewis esophagectomy with 2-field standard lymph-node dissection, McKeown esophagectomy with 3-field standard lymph-node dissection, for either ESCC (*N* = 6) or middle/lower EAC or EAC-EGJ Siewert I/II (*N* = 61). *N* = 21 patients with EAC-EGJ Siewert III adenocarcinoma were submitted to total extended gastrectomy. *N* = 7 EAC patients with small tumors extending borderline between EGJ Siewert II and III (with epicenter at 2 cm) were also submitted to total extended gastrectomy.

Within 90 days after surgery, post-operative recovery was uneventful in Ν = 48 (50.5%) patients. *N* = 23/95 (24.2%) patients developed Minor-Class II, whereas *N* = 24/95 (25.3%) developed Major-Class III-V complications including anastomotic leak in 7/95, conduit necrosis in 1/95 and tracheoesophageal fistula in 1/95 patients. Overall, in- and out-of-hospital 90-day mortality was 7.4% (*N* = 7/95).

In final histopathological examination, High Grade Dysplasia was noted in *N* = 2 (2.1%) patients with underlying Barret’s Esophagus, whereas invasive carcinomas encompassed Adenocarcinoma (*N* = 84, 88.4%), Adeno-squamous (*N* = 02, 2.1%), Squamous Cell Carcinoma (*N* = 06, 6.3%) and Mixed adeno-neuroendocrine carcinoma-MANEC (*N* = 01, 1.1%). Invasive tumors were well-differentiated in 2/95, moderately-differentiated in 36/95 and poorly-differentiated in 52/95 patients, while in *N* = 5 differentiation could not be assessed (Gx) or was not applicable (N/a).

In terms of residual disease, adequate resection margins were achieved in 86/95 (90.5%) and LN harvest > 15 nodes in 87/95 (91.6%) patients.

Long-term follow-up was completed in *N* = 92/95 patients (97%). Follow-up period ranged between 4 and 97 months with median 75 months for living and 20 months for deceased cases. During follow-up, two patients suffered myocardial infarction and one major UGI hemorrhage and passed away at 4th postoperative month. Additionally, one patient died because of myocardial infarction at 56th postoperative month and one died because of metachronous lung cancer disease progression at 74th postoperative month. *N* = 49/95 patients (51.6%) developed disease recurrence with *N* = 46/95 having passed away 4–87 months post-operatively. The remainder three patients developed lung metastasis (*N* = 2) and regional LN metastasis (*N* = 1) at 3, 17 and 36 months respectively. After treated with chemoradiotherapy and immunotherapy, all three are alive with stable disease (survival range 59–79 months). Thirty-one patients (32.6%) are alive and cancer-free with median survival of 77 months (range 58–97). In total, estimated median OS was 32.5 months (range: 4–97 months), while median DFS was 18.4 months (range: 2–97).

### Allele frequencies and genotype distributions reflecting the association between HOTAIR, LINC00951, POLR2E and HULC polymorphisms and cancer risk in EC, EAC, EAC-EGJ and ESCC Populations

For HOTAIR (Table 3), the detection of rs920778, C > T (T/C) polymorphism was initially performed in 95 surgically treated EC patients and 121 healthy controls with an overall distribution not significantly different between the two groups. According to our statistical analysis, the CT genotype was found to be equally present in both EC and control groups whereas the TT was overrepresented in the EC group with OR = 2.960, yet without statistical significance (*P* = 0.1241). When correlated EC patients’ TNM Stage and LN Involvement (N Status), we detected no significant association. When conducted a subset analysis by histological type, we identified a borderline non-significant increased frequency of the TT (OR: 3.442, *P* = 0.0620) and T variants (OR: 1.542, *P* = 0.0627) in *N* = 84 EAC patients. We then performed a subset analysis based on tumor location which yielded a significant over-presentation of the TT genotype (OR: 4.177, *P* = 0.0382) in *N* = 61 EAC-EGJ Siewert I/II patients followed by a marginally not significant over-presentation of the T allele (OR: 1.630, *P* = 0.0690). In *N* = 21 EAC-EGJ Siewert III and *N* = 6 ESCC patients, no correlation was found between TT and T gene variants and cancer susceptibility.

**Table 3:** Allele frequencies and genotype distributions demonstrating the association between HOTAIR polymorphism and cancer risk in Esophageal Cancer (EC), Esophageal AdenoCarcinoma (EAC), EsophagoGastric Junction (EGJ) Adenocarcinoma and Esophageal Squamous Cell Carcinoma (ESCC) Populations-Bold values denote statistically significant associations

Genotype: HOTAIR-SNP: rs920778, C > T (T/C)				
EC population (*N* = 95)	Surgical Case Group, *N* = 95 (%)	Control Group, *n* = 121 (%)	OR (95% CI)	P value
CC CT TT C allele T allele	50 (52.7) 37 (38.9) 8 (8.4) 137 (72.2) 53 (27.8)	74 (61.2) 43 (35.5) 4 (3.3) 191 (79) 51 (21)	1.00 (Ref.) 1.273 (0.7220–2.246) 2.960 (0.8455–10.363) 1.00 (Ref.) 1.449 (0.9305–2.256)	1.00 (Ref.) 0.4688 0.1241 1.00 (Ref.) 0.1127
EC: Stage (Pathological TNM), *N* = 95	I-II (*N* = 32) (%)	III-IV (*N* = 63) (%)	OR (95% CI)	P value
CC CT TT C allele T allele	17 (53.1) 12 (37.5) 3 (9.4) 46 (71.9) 18 (28.1)	33 (52.4) 25 (39.7) 5 (7.9) 91 (72.3) 35 (27.7)	1.00 (Ref.) 1.073 (0.4348–2.649) 0.8586 (0.1828–4.032) 1.00 (Ref.) 0.9829 (0.5029–1.921)	1.00 (Ref.) 1.0000 1.0000 1.00 (Ref.) 1.0000
EC: Lymph Node Involvement (N Status), *N* = 95	Negative (N0, *N* = 29) (%)	Positive (*N* > 1, *N* = 66) (%)	OR (95% CI)	P value
CC CT TT C allele T allele	16 (55.2) 11 (37.9) 2 (6.9) 43 (74.2) 15 (25.8)	34 (51.5) 26 (39.4) 6 (9.1) 94 (71.2) 38 (28.8)	1.00 (Ref.) 1.112 (0.4423–2.797) 1.412 (0.2560–7.786) 1.00 (Ref.) 1.159 (0.5765-2.330)	1.00 (Ref.) 1.0000 1.0000 1.00 (Ref.) 0.7282
EC: Disease Progression* (during follow up), *N* = 85/95 **	Negative (*N* = 36) (%)	Positive (*N* = 49) (%)	OR (95% CI)	P value
CC CT TT C allele T allele	17 (47.2) 14 (38.9) 5 (13.9) 48 (66.7) 24 (33.3)	25 (51) 22 (44.9) 2 (4.1) 72 (73.5) 26 (26.5)	1.00 (Ref.) 1.069 (0.4299–2.656) 0.2720 (0.04716 -1.569) 1.00 (Ref.) 0.7222 (0.3716–1.403)	1.00 (Ref.) 1.0000 0.2192 1.00 (Ref.) 0.3952
EAC subpopulation (*N* = 84/95)	Surgical Case Group, *N* = 84 (%)	Control Group, *n* = 121 (%)	OR (95% CI)	P value
CC CT TT C allele T allele	43 (51.2) 33 (39.3) 8 (9.5) 119 (70.9) 49 (29.1)	74 (61.2) 43 (35.5) 4 (3.3) 191 (78.9) 51 (21.1)	1.00 (Ref.) 1.321 (0.7327–2.381) 3.442 (0.9782–12.110) 1.00 (Ref.) 1.542 (0.9793–2.428)	1.00 (Ref.) 0.3694 0.0620 1.00 (Ref.) 0.0627
EAC located in EGJ subpopulation (*N* = 82/95)	EAC EGJ Siewert I-II, *N* = 61 (%)	Control Group, *n* = 121 (%)	OR (95% CI)	P value
CC CT TT C allele T allele	31 (50.8) 23 (37.7) 7 (11.5) 85 (69.7) 37 (30.3)	74 (61.2) 43 (35.5) 4 (3.3) 191 (78.9) 51 (21.1)	1.00 (Ref.) 1.277 (0.6615–2.464) **4.177 (1.140 to 15.303)** 1.00 (Ref.) 1.630 (0.9942–2.673)	1.00 (Ref.) 0.5016 **0.0382** 1.00 (Ref.) 0.0690
EAC located in EGJ subpopulation (*N* = 82/95)	EAC EGJ Siewert III, *N* = 21 (%)	Control Group, *n* = 121 (%)	OR (95% CI)	P value
CC CT TT C allele T allele	11 (52.4) 9 (42.9) 1 (4.7) 31 (73.9) 11 (26.1)	74 (61.2) 43 (35.5) 4 (3.3) 191 (79) 51 (21)	1.00 (Ref.) 1.408 (0.5402–3.670) 1.682 (0.1718–16.468) 1.00 (Ref.) 1.329 (0.6251–2.825)	1.00 (Ref.) 0.6188 0.5196 1.00 (Ref.) 0.5435
ESCC Subpopulation (*N* = 6/95)	ESCC, *N* = 6 (%)	Control Group, *n* = 121 (%)	OR (95% CI)	P value
CC CT TT C allele T allele	4 (66.7) 2 (33.3) 0 (0) 10 (83.4) 2 (16.6)	74 (61.2) 43 (35.5) 4 (3.3) 191 (79) 51 (21)	1.00 (Ref.) 0.8605 (0.1512–4.897) 1.840 (0.08509–39.766) 1.00 (Ref.) 0.7490 (0.1590–3.528)	1.00 (Ref.) 1.0000 1.0000 1.00 (Ref.) 1.0000

Notes: * Disease Progression including Any or Combination of: Local Recurrence, Regional LN Metastasis or Distant Metastasis, ** *N* = 85/95, excluding *N* = 3/95 Lost in Follow-up and *N* = 7/95 with 90-day in-hospital postoperative mortality

Regarding LINC00951 (Table 4), we explored the LINC00951 rs11752942, A > G (G/A) polymorphism prevalence in both cancer and healthy control groups. Overall, the distribution of LINC00951 rs11752942, A > G (G/A) genotype frequencies between EC patients and controls did not differ significantly. Based on our analysis, the AG genotype was equally distributed between the EC patients and community controls while the GG was not significantly less frequent in the surgical case group (OR: 0.5222, *P* = 0.2498). When assessing the relationship with patients’ TNM Stage and LN Involvement our analysis resulted in no association, although the GG genotype were detected more often in individuals in more advanced stages III-IV compared with the I-II subgroup (OR: 4.250, *P* = 0.2402) as observed also in the recurrence positive group compared with the disease free group (OR: 3.161, *P* = 0.4153). By performing subgroup analysis, we demonstrated that GG genotype may act as a protective factor in EAC (OR: 0.6026), EAC-EGJ Siewert I/II (OR: 0.3917) and ESCC (OR: 0.5758), whereas on the contrary it may pose a risk-factor in EAC-EGJ Siewert III patients (OR: 1.306). Nevertheless, none of the above associations were found statistically significant.

**Table 4:** Allele frequencies and genotype distributions demonstrating the association between LINC00951 polymorphism and cancer risk in Esophageal Cancer (EC), Esophageal AdenoCarcinoma (EAC), EsophagoGastric Junction (EGJ) Adenocarcinoma and Esophageal Squamous Cell Carcinoma (ESCC) Populations-Bold values denote statistically significant associations

Genotype: LINC00951 SNP: rs11752942, A > G (G/A)				
EC population (*N* = 95)	Surgical Case Group, *N* = 95 (%)	Control Group, *n* = 121 (%)	OR (95% CI)	P value
AA AG GG A allele G allele	45 (47.4) 42 (44.2) 8 (8.4) 132 (69.5) 58 (30.5)	47 (38.9) 58 (47.9) 16 (13.2) 152 (62.9) 90 (37.1)	1.00 (Ref.) 0.7563 (0.4277–1.337) 0.5222 (0.2035-1.340) 1.00 (Ref.) 0.7421 (0.4954–1.112)	1.00 (Ref.) 0.3847 0.2498 1.00 (Ref.) 0.1541
EC: Stage (Pathological TNM), *N* = 95	I-II (*N* = 32) (%)	III-IV (*N* = 63) (%)	OR (95% CI)	P value
AA AG GG A allele G allele	17 (53.1) 14 (43.8) 1 (3.1) 48 (75) 16 (25)	28 (44.4) 28 (44.4) 7 (11.2) 84 (66.6) 42 (33.4)	1.00 (Ref.) 1.214 (0.5035–2.929) 4.250 (0.4801–37.626) 1.00 (Ref.) 1.500 (0.7627-2.950)	1.00 (Ref.) 0.8230 0.2402 1.00 (Ref.) 0.2496
EC: Lymph Node Involvement (N Status), *N* = 95	Negative (N0, *N* = 29) (%)	Positive (*N* > 1, *N* = 66) (%)	OR (95% CI)	P value
AA AG GG A allele G allele	14 (48.3) 14 (48.3) 1 (3.4) 42 (72.5) 16 (27.5)	31 (47) 28 (42.4) 7 (10.6) 90 (68.2) 42 (31.8)	1.00 (Ref.) 0.9032 (0.3672–2.222) 3.161 (0.3542–28.213) 1.00 (Ref.) 1.225 (0.6190–2.424)	1.00 (Ref.) 1.0000 0.4153 1.00 (Ref.) 0.6108
EC; Disease Progression* (during follow up), *N* = 85/95 **	Negative (*N* = 36) (%)	Positive (*N* = 49) (%)	OR (95% CI)	P value
AA AG GG A allele G allele	15 (41.7) 18 (50) 3 (8.3) 48 (66.7) 24 (33.3)	25 (51) 19 (38.8) 5 (10.2) 69 (70.4) 29 (29.6)	1.00 (Ref.) 0.6333 (0.2553–1.571) 1.000 (0.2084– 4.799) 1.00 (Ref.) 0.8406 (0.4368–1.618)	1.00 (Ref.) 0.3631 1.0000 1.00 (Ref.) 0.6189
EAC subpopulation (*N* = 84/95)	Surgical Case Group, *N* = 84 (%)	Control Group, *n* = 121 (%)	OR (95% CI)	P value
AA AG GG A allele G allele	39 (46.4) 37 (44.1) 8 (9.5) 115 (68.5) 53 (31.5)	47 (38.8) 58 (48) 16 (13.2) 152 (62.8) 90 (37.2)	1.00 (Ref.) 0.7688 (0.4253–1.390) 0.6026 (0.2332–1.557) 1.00 (Ref.) 0.7784 (0.5129–1.181)	1.00 (Ref.) 0.4512 0.3547 1.00 (Ref.) 0.2483
EAC located in EGJ subpopulation (*N* = 82/95)	EAC EGJ Siewert I-II, *N* = 61 (%)	Control Group, *n* = 121 (%)	OR (95% CI)	P value
AA AG GG A allele G allele	30 (49.2) 27 (44.3) 4 (6.5) 87 (71.4) 35 (28.6)	47 (38.9) 58 (47.9) 16 (13.2) 152 (62.9) 90 (37.1)	1.00 (Ref.) 0.7293 (0.3819–1.393) 0.3917 (0.1194–1.285) 1.00 (Ref.) 0.6794 (0.4242–1.088)	1.00 (Ref.) 0.4105 0.1873 1.00 (Ref.) 0.1284
EAC located in EGJ subpopulation (*N* = 82/95)	EAC EGJ Siewert III, *N* = 21 (%)	Control Group, *n* = 121 (%)	OR (95% CI)	P value
AA AG GG A allele G allele	9 (42.9) 8 (38.1) 4 (19) 26 (62) 16 (38)	47 (38.9) 58 (47.9) 16 (13.2) 152 (62.9) 90 (37.1)	1.00 (Ref.) 0.7203 (0.2578–2.012) 1.306 (0.3531–4.827) 1.00 (Ref.) 1.039 (0.5290–2.042)	1.00 (Ref.) 0.6046 0.7342 1.00 (Ref.) 1.0000
ESCC Subpopulation (*N* = 6/95)	ESCC, *N* = 6 (%)	Control Group, *n* = 121 (%)	OR (95% CI)	P value
AA AG GG A allele G allele	2 (33.3) 4 (66.7) 0 (0) 8 (66.7) 4 (33.3)	47 (38.9) 58 (47.9) 16 (13.2) 152 (62.9) 90 (37.1)	1.00 (Ref.) 1.621 (0.2842–9.241) 0.5758 (0.02624–12.633) 1.00 (Ref.) 0.8444 (0.2472–2.885)	1.00 (Ref.) 0.6923 1.0000 1.00 (Ref.) 1.0000

Notes: * Disease Progression including Any or Combination of: Local Recurrence, Regional LN Metastasis or Distant Metastasis, ** *N* = 85/95, excluding *N* = 3/95 Lost in Follow-up and *N* = 7/95 with 90-day in-hospital postoperative mortality

We also evaluated the distribution of POLR2E rs3787016, T > C (C/T) polymorphism in surgical cancer and healthy control groups (Table 5). We detected both the C allele and the CC genotype less frequently in EC patients compared with the healthy controls yet marginally not significantly different (*P* = 0.0561, *P* = 0.0582 respectively). However, when evaluated gene frequencies based on the TNM stage, the CC variant was significantly underrepresented in individuals at more advanced stages of the disease: 2/30 patients in the III-IV Stage Subgroup compared with 7/14 patients in the I-II Stage Subgroup, OR = 0.1333 (95% CI: 0.02448–0.7263), *P* = 0.0209 indicating a possible protective role in disease burden genetic footprint. When assessing POLR2E rs3787016’s correlation with the LN Involvement or with the disease progression/recurrence risk, our univariate analysis demonstrated no statistically significant association, nonetheless the CC/TT genotypes were under-represented in the more advanced *N* > 1 stage compared with the N0 subgroup (OR = 0.2333, *P* = 0.0666) as well as in the recurrence positive group compared with the disease free group (OR = 0.7083, *P* = 0.7102). Subsequently our subgroup analysis assessing pure EAC population, detected the C allele significantly more frequent in the healthy controls compared with the EAC (OR: 0.5778, *P* = 0.0119) or with the EAC-EGJ Siewert I/II populations (OR: 0.6047, *P* = 0.0386). The CC genotype was also significantly more present in the healthy control population compared with either the EAC (OR: 0.2497, *P* = 0.0114) or the EAC-EGJ Siewert I/II populations (OR: 0.2194, *P* = 0.0220). While the C and the CC variants may hence represent a potential protective factor in esophageal adenocarcinoma/EGJ carcinogenesis pathway, no association was identified with the EAC Siewert III subgroup. By contrast, both C and CC variants were observed more frequently in the ESCC group indicating that may pose a risk-factor in ESCC aetiopathogenesis, yet the sample-size is too small (*N* = 6 patients) to extract safe results.

**Table 5:** Allele frequencies and genotype distributions demonstrating the association between POL2RE polymorphism and cancer risk in Esophageal Cancer (EC), Esophageal AdenoCarcinoma (EAC), EsophagoGastric Junction (EGJ) Adenocarcinoma and Esophageal Squamous Cell Carcinoma (ESCC) Populations-Bold values denote statistically significant associations

Genotype: POLR2E SNP: rs3787016, T > C (C/T)				
EC population (*N* = 95)	Surgical Case Group, *N* = 95 (%)	Control Group, *n* = 121 (%)	OR (95% CI)	P value
TT CT CC T allele C allele	44 (46.3) 42 (44.2) 9 (9.5) 130 (68.4) 60 (31.6)	43 (35.6) 57 (47.1) 21 (17.3) 143 (59.2) 99 (40.8)	1.00 (Ref.) 0.7201 (0.4034–1.285) 0.4188 (0.1725–1.017) 1.00 (Ref.) 0.6667 (0.4473–0.9937)	1.00 (Ref.) 0.3033 0.0582 1.00 (Ref.) 0.0561
EC: Stage (Pathological TNM), *N* = 95	I-II (*N* = 32) (%)	III-IV (*N* = 63) (%)	OR (95% CI)	P value
TT CT CC T allele C allele	14 (43.8) 11 (34.4) 7 (21.8) 39 (61) 25 (39)	30 (47.6) 31 (49.2) 2 (3.2) 91 (72.2) 35 (27.8)	1.00 (Ref.) 1.315 (0.5158–3.353) **0.1333 (0.02448–0.7263)** 1.00 (Ref.) 0.600 (0.3177–1.133)	1.00 (Ref.) 0.6385 **0.0209** 1.00 (Ref.) 0.1376
EC: Lymph Node Involvement (N Status), *N* = 95	Negative (N0, *N* = 29) (%)	Positive (*N* > 1, *N* = 66) (%)	OR (95% CI)	P value
TT CT CC T allele C allele	14 (48.3) 9 (31) 6 (20.7) 37 (63.8) 21 (36.2)	30 (45.5) 33 (50) 3 (4.5) 93 (70.5) 39 (29.5)	1.00 (Ref.) 1.711 (0.6469–4.526) 0.2333 (0.05080–1.072) 1.00 (Ref.) 0.7389 (0.3845-1.420)	1.00 (Ref.) 0.3340 0.0666 1.00 (Ref.) 0.3987
EC: Disease Progression* (during follow up), *N* = 85/95 **	Negative (*N* = 36) (%)	Positive (*N* = 49) (%)	OR (95% CI)	P value
TT CT CC T allele C allele	17 (47.2) 15 (41.7) 4 (11.1) 49 (68.1) 23 (31.9)	24 (49) 21 (42.9) 4 (8.1) 69 (70.5) 29 (29.5)	1.00 (Ref.) 0.9917 (0.3997–2.460) 0.7083 (0.1550–3.236) 1.00 (Ref.) 0.8954 (0.4634-1.730)	1.00 (Ref.) 1.0000 0.7102 1.00 (Ref.) 0.7399
EAC subpopulation (*N* = 84/95)	Surgical Case Group, *N* = 84 (%)	Control Group, *n* = 121 (%)	OR (95% CI)	P value
TT CT CC T allele C allele	41 (48.8) 38 (45.2) 5 (6) 120 (71.4) 48 (28.6)	43 (35.5) 57 (47.1) 21 (17.4) 143 (59.1) 99 (40.9)	1.00 (Ref.) 0.6992 (0.3864–1.265) **0.2497 (0.08606–0.7246)** 1.00 (Ref.) **0.5778 (0.3790–0.8808)**	1.00 (Ref.) 0.2912 **0.0114** 1.00 (Ref.) **0.0119**
EAC located in EGJ subpopulation (*N* = 82/95)	EAC EGJ Siewert I-II, *N* = 61 (%)	Control Group, *n* = 121 (%)	OR (95% CI)	P value
TT CT CC T allele C allele	28 (45.9) 30 (49.2) 3 (4.9) 86 (70.5) 36 (29.5)	43 (35.5) 57 (47.1) 21 (17.4) 143 (59.1) 99 (40.9)	1.00 (Ref.) 0.8083 (0.4221–1.548) **0.2194 (0.05977–0.8052)** 1.00 (Ref.) **0.6047 (0.3794–0.9636)**	1.00 (Ref.) 0.6189 **0.0220** 1.00 (Ref.) **0.0386**
EAC located in EGJ subpopulation (*N* = 82/95)	EAC EGJ Siewert III, *N* = 21 (%)	Control Group, *n* = 121 (%)	OR (95% CI)	P value
TT CT CC T allele C allele	12 (57.1) 7 (33.3) 2 (9.6) 31 (73.8) 11 (26.2)	43 (35.5) 57 (47.1) 21 (17.4) 143 (59.1) 99 (40.9)	1.00 (Ref.) 0.4401 (0.1598–1.212) 0.3413 (0.06990–1.666) 1.00 (Ref.) 0.5125 (0.2460–1.068)	1.00 (Ref.) 0.1343 0.2110 1.00 (Ref.) 0.0862
ESCC Subpopulation (*N* = 6/95)	ESCC, *N* = 6 (%)	Control Group, *n* = 121 (%)	OR (95% CI)	P value
TT CT CC T allele C allele	1 (16.7) 2 (33.3) 3 (50) 4 (33.4) 8 (66.6)	43 (35.6) 57 (47.1) 21 (17.3) 143 (59.2) 99 (40.8)	1.00 (Ref.) 1.509 (0.1324–17.198) 6.143 (0.6018–62.703) 1.00 (Ref.) 2.889 (0.8464-9.860)	1.00 (Ref.) 1.0000 0.1224 1.00 (Ref.) 0.1311

Notes: * Disease Progression including Any or Combination of: Local Recurrence, Regional LN Metastasis or Distant Metastasis, ** *N* = 85/95, excluding *N* = 3/95 Lost in Follow-up and *N* = 7/95 with 90-day in-hospital postoperative mortality

Finally, we investigated HULC rs7763881 incidence as per previous methodology principles (Table 6). Overall, the distribution of HULC rs7763881, A > C (C/A) among EC and control populations was not found different, with both AC and CC genotypes being equally present not only in EC patients but also in the healthy controls, same as the C allele. Concurrently, we identified no association of the HULC rs7763881 with any of the clinical variables in EC patients such as TNM stage and LN Involvement. The subset analysis by tumor histology and location yielded also no association with cancer susceptibility in none of the AC/AA, CC/AA and C/A genetic models with no significant variations among ESCC, EAC, EAC-EGJ Siewert I/II and EGJ Siewert III subpopulations.

**Table 6:** Allele frequencies and genotype distributions demonstrating the association between HULC polymorphism and cancer risk in Esophageal Cancer (EC), Esophageal AdenoCarcinoma (EAC), EsophagoGastric Junction (EGJ) Adenocarcinoma and Esophageal Squamous Cell Carcinoma (ESCC) Populations-Bold values denote statistically significant associations

Genotype: HULC SNP: rs7763881, A > C (C/A)				
EC population (*N* = 95)	Surgical Case Group, *N* = 95 (%)	Control Group, *n* = 121 (%)	OR (95% CI)	P value
AA AC CC A allele C allele	30 (31.6) 42 (44.2) 23 (24.2) 102 (53.7) 88 (46.3)	35 (28.9) 63 (52.1) 23 (19) 133 (55) 109 (45)	1.00 (Ref.) 0.7778 (0.4164–1.453) 1.167 (0.5476–2.486) 1.00 (Ref.) 1.053 (0.7189–1.542)	1.00 (Ref.) 0.5231 0.7044 1.00 (Ref.) 0.8458
EC: Stage (Pathological TNM), *N* = 95	I-II (*N* = 32) (%)	III-IV (*N* = 63) (%)	OR (95% CI)	P value
AA AC CC A allele C allele	9 (28.1) 15 (46.9) 8 (25) 33 (51.6) 31 (48.4)	21 (33.3) 27 (42.9) 15 (23.8) 69 (54.8) 57 (45.2)	1.00 (Ref.) 0.7714 (0.2826–2.106) 0.8036 (0.2518–2.565) 1.00 (Ref.) 0.8794 (0.4812–1.607)	1.00 (Ref.) 0.8002 0.7720 1.00 (Ref.) 0.7585
EC: Lymph Node Involvement (N Status), *N* = 95	Negative (N0, *N* = 29) (%)	Positive (*N* > 1, *N* = 66) (%)	OR (95% CI)	P value
AA AC CC A allele C allele	10 (34.5) 11 (37.9) 8 (27.6) 31 (53.5) 27 (46.5)	20 (30.3) 31 (47) 15 (22.7) 71 (53.8) 61 (46.2)	1.00 (Ref.) 1.409 (0.5058 to 3.926) 0.9375 (0.2981–2.949) 1.00 (Ref.) 0.9864 (0.5310–1.832)	1.00 (Ref.) 0.6020 1.0000 1.00 (Ref.) 1.0000
EC: Disease Progression* (during follow up), *N* = 85/95 **	Negative (*N* = 36) (%)	Positive (*N* = 49) (%)	OR (95% CI)	P value
AA AC CC A allele C allele	11 (30.6) 16 (44.4) 9 (25) 38 (52.8) 34 (47.2)	14 (28.6) 21 (42.9) 14 (28.5) 49 (50.1) 49 (49.9)	1.00(Ref.) 1.031 (0.3706–2.869) 1.222 (0.3865–3.865) 1.00 (Ref.) 1.118 (0.6078–2.055)	1.00(Ref.) 1.0000 0.7765 1.00 (Ref.) 0.7577
EAC subpopulation (*N* = 84/95)	Surgical Case Group, *N* = 84 (%)	Control Group, *n* = 121 (%)	OR (95% CI)	P value
AA AC CC A allele C allele	26 (31) 37 (44) 21 (25) 89 (53) 79 (47)	35 (28.9) 63 (52.1) 23 (19) 133 (55) 109 (45)	1.00 (Ref.) 0.7906 (0.4127–1.514) 1.229 (0.5637–2.680) 1.00 (Ref.) 1.083 (0.7297–1.608)	1.00 (Ref.) 0.5086 0.6919 1.00 (Ref.) 0.7625
EAC located in EGJ subpopulation (*N* = 82/95)	EAC EGJ Siewert I-II, *N* = 61 (%)	Control Group, *n* = 121 (%)	OR (95% CI)	P value
AA AC CC A allele C allele	19 (31.1) 26 (42.7) 16 (26.2) 64 (52.5) 58 (47.5)	35 (28.9) 63 (52.1) 23 (19) 133 (55) 109 (45)	1.00 (Ref.) 0.7602 (0.3694–1.565) 1.281 (0.5487–2.993) 1.00 (Ref.) 1.106 (0.7147–1.711)	1.00 (Ref.) 0.4641 0.6655 1.00 (Ref.) 0.6576
EAC located in EGJ subpopulation (*N* = 82/95)	EAC EGJ Siewert III, *N* = 21 (%)	Control Group, *n* = 121 (%)	OR (95% CI)	P value
AA AC CC A allele C allele	7 (33.3) 9 (42.9) 5 (23.8) 23 (54.8) 19 (45.2)	35 (28.9) 63 (52.1) 23 (19) 133 (55) 109 (45)	1.00 (Ref.) 0.7143 (0.2448–2.084) 1.087 (0.3075–3.843) 1.00 (Ref.) 1.008 (0.5218–1.947)	1.00 (Ref.) 0.5825 1.0000 1.00 (Ref.) 1.0000
ESCC Subpopulation (*N* = 6/95)	ESCC, *N* = 6 (%)	Control Group, *n* = 121 (%)	OR (95% CI)	P value
AA AC CC A allele C allele	1 (16.7) 4 (66.6) 1 (16.7) 6 (50) 6 (50)	35 (28.9) 63 (52.1) 23 (19) 133 (55) 109 (45)	1.00 (Ref.) 2.222 (0.2388–20.676) 1.522 (0.09052–25.581) 1.00 (Ref.) 1.220 (0.3826–3.892)	1.00 (Ref.) 0.6552 1.0000 1.00 (Ref.) 0.7735

Notes: * Disease Progression including Any or Combination of: Local Recurrence, Regional LN Metastasis or Distant Metastasis, ** *N* = 85/95, excluding *N* = 3/95 Lost in Follow-up and *N* = 7/95 with 90-day in-hospital postoperative mortality

### Univariate and Multivariate Survival Analysis reflecting the association of HOTAIR, LINC00951, POLR2E and HULC polymorphisms with overall survival (OS) and Disease-Free Survival (DFS) in EC, EAC, EAC-EGJ and ESCC Populations

Univariate analysis of the independent variables did not reveal any significant predictors for death or recurrence in both whole EC population and subpopulations for none of the four SNPs of interest. Subsequent multivariate analysis included univariate predictors of age, stage, operation type, histological subtype, neo- and adjuvant chemotherapy, radiotherapy and resection status. When performed for the whole EC cohort (*N* = 95), multivariate analysis revealed significant worse OS associated with age (HR: 1.0356, *P* = 0.0299), stage III-IV (OR: 3.9017, *P* = 0.0017), ESCC (HR: 4.1507, *P* = 0.04951), whereas only stage III-IV was associated with worse DFS (HR: 4.0091, *P* = 0.0047). When performed for either full EC cohort or subpopulations, no risk association was demonstrated between OS or DFS and the occurrence of any of the studied SNPs gene variants (Online Resource 1: Tables 1, 2, 3, 4 and 5; Figs. 1, 2 and 3).

## Discussion

Human genome sequencing has established that protein-coding genes account for 3% of DNA whilst over 80% of our genome is actively transcribed into a group of a noncoding RNA molecules (ncRNAs) without potentiality for protein-coding, including lncRNAs [28]. These transcripts play a pivotal role in a series of biological processes as regulating chromatin dynamics, genome packaging and neighboring protein gene expression, growth and differentiation and hence implicated in carcinogenesis [29]. SNPs are the most common genetic variation and these occurring in lncRNAs’ functional region largely affect their expression, structure, and function and thereby affect cancer prognosis [30] and susceptibility by promoting oncogenesis and influencing disease-recurrence risk [31]. As such, lncRNAs SNPs hold great potential not only as future therapeutic targets/agents but also as novel markers for predictive analysis of cancer risk, clinical outcome, prognosis, survival and drug resistance [32] or toxicity [33].

The development of EC is a multifactorial process and associations with genetic variants have already been identified in Asian studies [34]. Considering that the majority of published research investigating this association of lncRNAs SNPs in esophageal carcinogenesis has been performed in Eastern ethnicities where ESCC histological subtype predominates, we hypothesized that likewise these SNPs may also manifest differently in EC carriers of western ethnicity with dominant EAC prevalence instead. To test this hypothesis, we investigated the effects of four lncRNAs polymorphisms on EC, EAC, EAC-EGJ and ESCC cancer susceptibility in a western population as Greece. We further sought to compare underlying correlations between Siewert I/II and Siewert III EGJ adenocarcinoma to identify possible variations in their oncogenetic mechanisms.

HOX transcript antisense RNA (HOTAIR) is a 2158-nucleotide lncRNA transcribed from the homeobox C (HOXC) antisense strand genes cluster located in chromosome 12q13.12 [35]. A growing number of investigations are drawing attention to the relationship between HOTAIR’s SNPs and the risk of various cancer types but the results obtained so far have been equivocal. In 2014, Zhang et al [36] examined the relationship between HOTAIR’s SNPs and ESCC predisposition and concluded that, compared with the rs920778 CC genotype, the TT played a positive role in the ESCC risk within a Chinese population. In 2021, Xu et al. also suggested that the T allele was nominally significant related to GC susceptibility among Chinese population when compared with the rs920778 C allele [37]. In 2017, Ge’s meta-analysis [38] comprising 37,900 samples from 26 case-control studies identified significant statistical evidence between the rs920778 and cancer susceptibility. When stratified by cancer type, a significantly increased susceptibility to ESCC and GC was also uncovered. By contrast, Bayram et al [39] concluded that HOTAIR rs920778 did not contribute to GC incidence in Turkish population. Taking into account previous evidence, we performed a case-control study analyzing the distribution of HOTAIR rs920778 genotype frequencies in both EC and healthy controls which yielded not significant over-presentation of the TT genotype in our EC population (OR: 2.960) encompassing various histological subtypes. However, in the subsequent subgroup analysis by histological type a significantly increased susceptibility to EAC-EGJ Siewert I/II cancer in the Greek population was uncovered in the homozygous TT models (OR: 4.177, *P* = 0.0382), suggesting that HOTAIR rs920778, C > T (T/C) polymorphism may pose a risk-factor in the aetiopathogenesis of the EAC in the West in a similar pattern as shown by previous studies for the ESCC carcinogenesis mechanism in the East [40, 41].

LINC00951 is a lncRNA located in chromosome 6p21.2, informally studied as lincRNA-uc003opf.1. Among 52 SNPs, Wu et al’s [42] genotyping results demonstrated that LINC00951 rs11752942 A > G (G/A) was significantly associated with ESCC risk. Pan et al [43] meta-analysis also identified that HOTAIR rs920778 and LINC00951 rs11752942 were related to head and neck cancers’ incidence in Asia. Taking these into consideration, we conducted a case-control study to determine possible association between this polymorphism and EC/EAC/ESCC risk in Greek population. As opposed to previous studies of Asian background, the distribution of LINC00951 rs11752942, A > G (G/A) genotype frequencies between EC patients and controls did not differ significantly in neither our primary analysis including all histological subtypes and tumor locations nor in our subgroup analysis investigating EAC, ESCC and EGJ Siewert I/II and III subsets. This outcome may plausibly be explained by the fact that the EAC subtype manifests predominantly in our EC patients of Greek ethnicity compared to ESCC subtype with different genetic footprint which predominantly manifests in Eastern studies of Asian ancestry instead.

Although the literature presents conflicting notions, SNP rs3787016 (A > G or complementary T > C, C/T), localized in the fourth intron of RNA polymerase II subunit E (POLR2E) lncRNA gene may serve as a genetic risk-factor increasing predisposition to various cancer types [41] including prostate cancer in Chinese [44] or Iranian [45], gastric cancer [46] in Chinese, breast and cervical cancer in Chinese populations [47]. Conversely, POLR2E rs3787016 may serve as a genetic protective factor against esophageal cancer ESCC subtype as investigated by Kang et al. in Chinese/Han population [17] in 2015. Since no study has yet evaluated the role of POLR2E rs3787016 in the diagnosis, incidence and prognosis of EC/EAC in the west, we conducted a case-control study in a Greek/European ancestry population. Our primary analysis yielded that CC genotype (OR: 0.4188) and C allele (OR: 0.6667) carriers are observed more frequently in healthy community controls and when present in EC patients are associated with lower LN infiltration risk (OR: 0.2333) and lesser metastatic potential (OR: 0.7083), yet without statistical significance. However, when TNM stage was assessed, CC genotype was significantly reduced in more advanced stages (OR: 0.1333, *P* = 0.0209). Furthermore in our subgroup analysis, compared with the TT and CT genotypes, CC significantly reduced the risk of EAC (OR: 0.2497, *P* = 0.0114) as well as the risk of EAC-EGJ Siewert I/II (OR: 0.2194, *P* = 0.0220), whereas no association was detected with the EAC-EGJ Siewert III risk. C allele’s carriers were also significantly associated with lower EAC (*P* = 0.0119) and EAC-EGJ Siewert I/II (*P* = 0.0386) risk. In line with Kang’s study, we identified that POLR2E rs3787016 may also serve as a genetic protective factor against esophageal cancer EAC subtype predisposition similarly to the ESCC subtype.

Apart from investigating POLR2E rs3787016’s potential correlation with ESCC susceptibility, Kang also assessed the HULC rs7763881, A > C (C/A) incidence in the same Chinese/Han population [17] concluding that HULC rs7763881 was a protective factor against ESCC among male, younger patients. Hepatocellular carcinoma up-regulated lncRNA (HULC) gene is located in chromosome 6p24.3 with two exons and 1638 bp length. In a 2022 meta-analysis the authors resulted that rs7763881 was associated with a decreased hepatocellular, colorectal and esophageal cancer risk [48]. Given literature’s contradictory results with Hong et al [49] suggesting that the HULC rs7763881 is associated with increased GC susceptibility and Elhelaly et al. [50]suggesting association with breast cancer in Egyptians, we conducted our case-control study to explore its role in EC/EAC/ESCC genetic footprint in western ethnicity. Compared with the previous studies, both our primary and subgroup analysis by tumor histology and location demonstrated no cancer susceptibility association in any of the genetic models AC/AA, CC/AA and C/A alleles with no significant variations among ESCC, EAC, EAC-EGJ Siewert I/II or EGJ-Siewert III subpopulations. This could be explained by small sample-size or EAC subtype’s predominant prevalence in our cohort or may represent a true different genetic footprint needs to be confirmed by additional future studies in the west.

Certain limitations apply to this report. As a hospital-based case-control study with large majority of EC cases and healthy controls from Attica Region, inherent choice bias may have occurred. A variability in sample-size was present related to histological subtypes and tumor location included in the primary analysis, which we sought to overcome with the subsequent subgroup analyses. The statistical strength of this case-control study may also be somewhat limited by the sample-size, particularly concerning the statistical analyses of EGJ Siewert I/II, Siewert III and ESCC subsets where the smaller sizes may have impacted the credibility of the data. Therefore, throughout the present analysis we intentionally focused on interpreting data involving larger sample-size groups such as EC or EAC rather than EGJ Siewert III or ESCC subgroups. Finally despite this case-control study is retrospective by definition, all data were extracted from our prospectively-collected UGI cancer database following predetermined research protocol to ensure appropriate methodology. Additional study-strengths were the follow-up length with high case-ascertainment enabling us to perform our correlations between SNPs and clinicopathological data such as tumor progression, metastasis and overall survival.

## Conclusion

Neither LINC00951 rs11752942 nor HULC rs7763881 polymorphisms were detected more frequently in surgically treated EC patients compared with healthy community subjects in our study. Regarding HOTAIR rs920778, our subgroup analysis findings indicate that TT genotype may serve as a potential therapeutic suppression target against EAC-EGJ Siewert I-II. We also identified that the presence of C allele, as well as CC genotype of POLR2E rs3787016 were detected more often in the control population, and when found in esophageal cancerous tissues were associated with earlier stages of the disease, as well as with minor lymph node involvement and lesser metastatic potential. Thus, our findings regarding POLR2E rs3787016 suggest that it may become a valuable biomarker to evaluate EC predisposition and predict response to treatment and prognosis. These results demonstrate that HOTAIR and POLR2E genetic variants may influence lncRNA regulation and as such, may explain a fraction of EC and EAC genetic basis. Clinical implications of these findings need to be verified with further prospective studies with larger sample-size.

## Electronic supplementary material

Below is the link to the electronic supplementary material.

Supplementary Material 1

## Data Availability

The data generated in our study are available upon request from the corresponding author.

## Abbreviations

CRM: Circumferential resection margins
DFS: Disease-free survival
EAC: Esophageal AdenoCarcinoma
EC: Esophageal Cancer
ESCC: Esophageal Squamous Cell Carcinoma
EGJ: EsophagoGastric Junction
FFPE: Formalin-Fixed Paraffin-Embedded
GC: Gastric Cancer
GERD: Gastroesophageal reflux disease
HRs: Hazard Ratios
LN: Lymph Node
LncRNAs: Long non-coding RNAs
NKUA: National and Kapodistrian University of Athens
ORs: Odds Ratios
OS: Overall survival
SNPs: Single-Nucleotide-Polymorphisms
UGI: Upper GastroIntestinal
