# Supplementary material for: Associations of long non-coding RNAs HOTAIR, LINC00951, POLR2E and HULC polymorphisms with the risk of esophageal and esophagogastric junction cancer in a western population: a case-control study
Source: Mol Biol Rep. 2024 Feb 1;51(1):249. doi: 10.1007/s11033-024-09206-0 (PMC10834655; doi:10.1007/s11033-024-09206-0)
Supplement: Supplementary file 1 — Supplementary Material 1 [file 11033_2024_9206_MOESM1_ESM.docx]

**Supplementary Material**

Table 1: Overall survival (OS) - Multivariate Analysis for the whole Esophageal Cancer (EC) cohort (N=95 patients) including the four SNPs of interest- Bold values denote statistically signiﬁcant associations.

| Median OS | 32.483 |  |  |  |  |  |
| --- | --- | --- | --- | --- | --- | --- |
| time | n.risk | n.event | survival | std.err | LCI | HCI |
| 0 | 92 | 0.000 | 1 | 0% | 1 | 1 |
| 72 | 27 | 55.000 | 0.402 | 0.051 | 0.313 | 0.516 |

| Multivariate Analysis | HR | LCI | HCI | P value |
| --- | --- | --- | --- | --- |
| Age | 1.0356 | 1.0034 | 1.069 | **0.02994** |
| Stage III-IV | 3.9017 | 1.665 | 9.143 | **0.00173** |
| Operative Technique: Total Gastrectomy | 0.5821 | 0.2786 | 1.217 | 0.15027 |
| Histological Subtype: ESCC | 4.1507 | 1.0031 | 17.176 | **0.04951** |
| Neoadjuvant Chemotherapy | 1.0605 | 0.5537 | 2.031 | 0.85946 |
| Adjuvant Chemotherapy | 1.0333 | 0.4708 | 2.268 | 0.93482 |
| Radiotherapy | 1.4304 | 0.6889 | 2.97 | 0.33696 |
| Resection Status: R1 | 2.2407 | 0.8706 | 5.767 | 0.09441 |
| CRM: Positive | 1.4749 | 0.606 | 3.59 | 0.39188 |
| HOTAIR CT | 0.6769 | 0.3506 | 1.307 | 0.24493 |
| HOTAIR TT | 0.5992 | 0.1961 | 1.831 | 0.36885 |
| LINC00951 AG | 0.5903 | 0.3122 | 1.116 | 0.10481 |
| LINC00951 GG | 0.3712 | 0.1179 | 1.169 | 0.09033 |
| POLR2E CT | 1.211 | 0.64066 | 2.2889 | 0.55569 |
| POLR2E CC | 1.5029 | 0.41585 | 5.4315 | 0.5343 |
| HULC AC | 0.9828 | 0.511 | 1.89 | 0.95852 |
| HULC CC | 0.6466 | 0.2907 | 1.438 | 0.28505 |

Notes: LCI: Lower Confidence Interval, HCI: Higher Confidence Interval, HR: Hazard Ratio, Stage: III-IV versus I-II, Operative Technique: Total Extended Gastrectomy versus Esophagectomy Group, Histological Subtype ESCC versus EAC, Adeno-squamous, MANEC, HGD, Resection Status: R1 versus R0, Circumferential Resection Margin (CRM).

Table 2: Disease- Free survival (DFS) - Multivariate Analysis for the whole Esophageal Cancer (EC) cohort (N=95 patients) including the four SNPs of interest- Bold values denote statistically signiﬁcant associations.

| Median DFS | 18.367 |  |  |  |  |  |
| --- | --- | --- | --- | --- | --- | --- |
| time | n.risk | n.event | survival | std.err | LCI | HCI |
| 0 | 91 | 0 | 1 | 0 | 1 | 1 |
| 72 | 23 | 48 | 0.407 | 0.0549 | 0.312 | 0.53 |

| Multivariate Analysis | HR | LCI | HCI | P value |
| --- | --- | --- | --- | --- |
| Age | 1.007 | 0.976 | 1.040 | 0.655 |
| Stage III-IV | 4.009 | 1.530 | 10.504 | **0.005** |
| Operative Technique: Total Gastrectomy | 0.769 | 0.346 | 1.708 | 0.519 |
| Histological Subtype: ESCC | 1.575 | 0.157 | 15.819 | 0.699 |
| Neoadjuvant Chemotherapy | 1.348 | 0.662 | 2.747 | 0.410 |
| Adjuvant Chemotherapy | 2.310 | 0.857 | 6.225 | 0.098 |
| Radiotherapy | 1.660 | 0.725 | 3.801 | 0.231 |
| Resection Status: R1 | 1.438 | 0.504 | 4.107 | 0.497 |
| CRM: Positive | 1.539 | 0.551 | 4.293 | 0.410 |
| HOTAIR CT | 0.846 | 0.414 | 1.729 | 0.647 |
| HOTAIR TT | 0.637 | 0.141 | 2.889 | 0.559 |
| LINC00951 AG | 0.570 | 0.264 | 1.232 | 0.153 |
| LINC00951 GG | 0.440 | 0.140 | 1.385 | 0.160 |
| POLR2E CT | 1.092 | 0.550 | 2.168 | 0.801 |
| POLR2E CC | 2.754 | 0.680 | 11.152 | 0.156 |
| HULC AC | 0.911 | 0.422 | 1.970 | 0.813 |
| HULC CC | 0.931 | 0.394 | 2.198 | 0.870 |

Notes: LCI: Lower Confidence Interval, HCI: Higher Confidence Interval, HR: Hazard Ratio, Stage: III-IV versus I-II, Operative Technique: Total Extended Gastrectomy versus Esophagectomy Group, Histological Subtype ESCC versus EAC, Adeno-squamous, MANEC, HGD, Resection Status: R1 versus R0, Circumferential Resection Margin (CRM).

Figure 1: Kaplan- Meier Curves for HOTAIR, LINC00951, POLR2E and HULC polymorphisms in Esophageal Cancer (EC) cohort


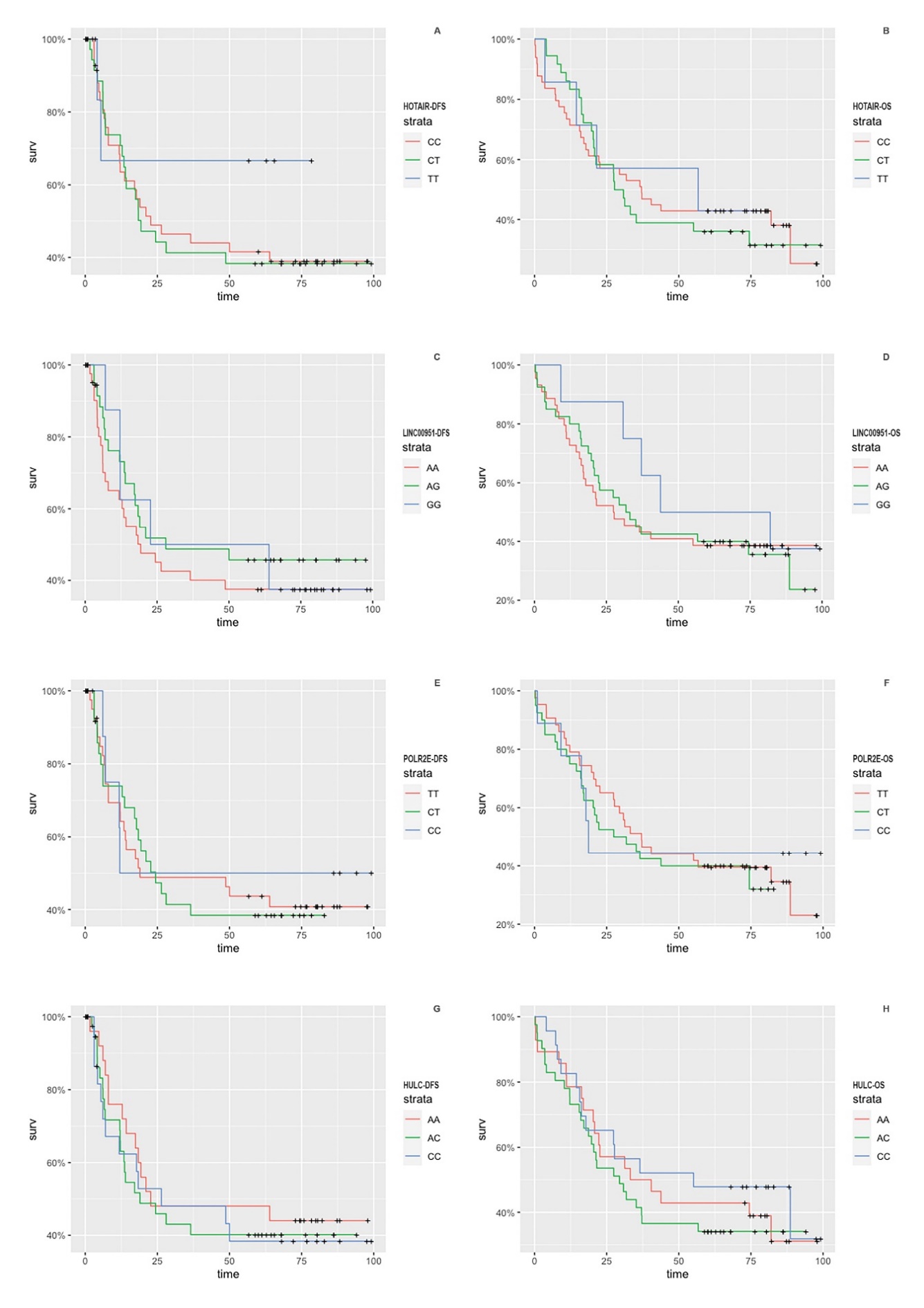


Note: A) HOTAIR-DFS, B) HOTAIR-OS, C) LINC00951-DFS, D) LINC00951-OS, E), POLR2E-DFS, F) POLR2E- OS, G) HULC-DFS, H) HULC-OS. No statistically signiﬁcant associations in either overall survival (OS) or Disease-Free Survival (DFS) analysis were demonstrated.

Figure 2: Multivariate Survival Analysis in Esophageal Squamous Cell Carcinoma (ESCC) subpopulation by Stage


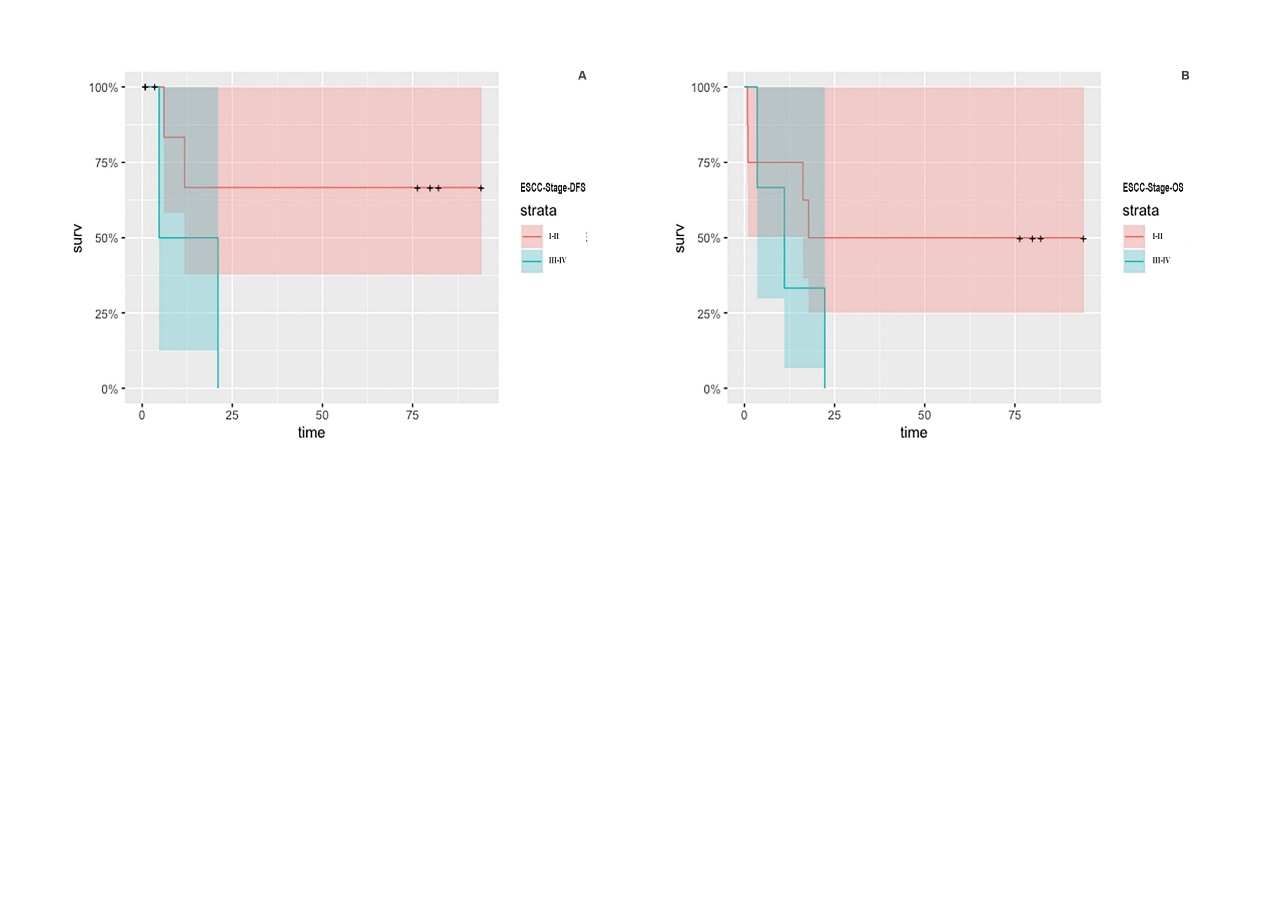


Table 3: Overall survival (OS) and Disease- Free survival (DFS)- Multivariate Analysis for the Esophageal AdenoCarcinoma (EAC) subpopulation (N=84/95 patients) including the four SNPs of interest- Bold values denote statistically signiﬁcant associations.

| OS Multivariate Analysis | HR | LCI | HCI | P value |
| --- | --- | --- | --- | --- |
| Age | 1.028 | 0.995 | 1.061 | 0.094 |
| Stage III-IV | 3.165 | 1.339 | 7.481 | **0.009** |
| Neoadjuvant Chemotherapy | 0.746 | 0.360 | 1.543 | 0.429 |
| Adjuvant Chemotherapy | 0.930 | 0.397 | 2.177 | 0.868 |
| Radiotherapy | 1.226 | 0.585 | 2.571 | 0.589 |
| Resection Status: R1 | 1.636 | 0.662 | 4.043 | 0.286 |
| CRM: Positive | 2.112 | 0.782 | 5.703 | 0.140 |
| HOTAIR CT | 1.196 | 0.633 | 2.257 | 0.582 |
| HOTAIR TT | 0.834 | 0.272 | 2.562 | 0.751 |
| LINC00951 AG | 0.664 | 0.349 | 1.263 | 0.212 |
| LINC00951 GG | 0.477 | 0.154 | 1.476 | 0.199 |
| POLR2E CT | 0.890 | 0.450 | 1.760 | 0.738 |
| POLR2E CC | 0.664 | 0.121 | 3.631 | 0.636 |
| HULC AC | 0.899 | 0.439 | 1.839 | 0.770 |
| HULC CC | 0.633 | 0.280 | 1.434 | 0.273 |

| DFS Multivariate Analysis | OR | LCI | HCI | P value |
| --- | --- | --- | --- | --- |
| Age | 1.008 | 0.975 | 1.042 | 0.635 |
| Stage III-IV | 3.798 | 1.395 | 10.340 | **0.009** |
| Neoadjuvant Chemotherapy | 1.211 | 0.566 | 2.591 | 0.622 |
| Adjuvant Chemotherapy | 2.189 | 0.788 | 6.085 | 0.133 |
| Radiotherapy | 1.379 | 0.608 | 3.127 | 0.442 |
| Resection Status: R1 | 1.322 | 0.467 | 3.744 | 0.599 |
| CRM: Positive | 1.543 | 0.508 | 4.689 | 0.444 |
| HOTAIR CT | 0.986 | 0.487 | 1.996 | 0.969 |
| HOTAIR TT | 0.612 | 0.134 | 2.786 | 0.525 |
| LINC00951 AG | 0.691 | 0.333 | 1.434 | 0.321 |
| LINC00951 GG | 0.577 | 0.188 | 1.771 | 0.337 |
| POLR2E CT | 1.016 | 0.503 | 2.056 | 0.964 |
| POLR2E CC | 1.286 | 0.232 | 7.128 | 0.773 |
| HULC AC | 0.934 | 0.407 | 2.145 | 0.872 |
| HULC CC | 0.882 | 0.360 | 2.163 | 0.784 |

Notes: LCI: Lower Confidence Interval, HCI: Higher Confidence Interval, HR: Hazard Ratio, Stage: III-IV versus I-II, Resection Status: R1 versus R0, Circumferential Resection Margin (CRM)

Figure 3: Multivariate Survival Analysis in Esophageal Adenocarcinoma (EAC) subpopulation by Stage


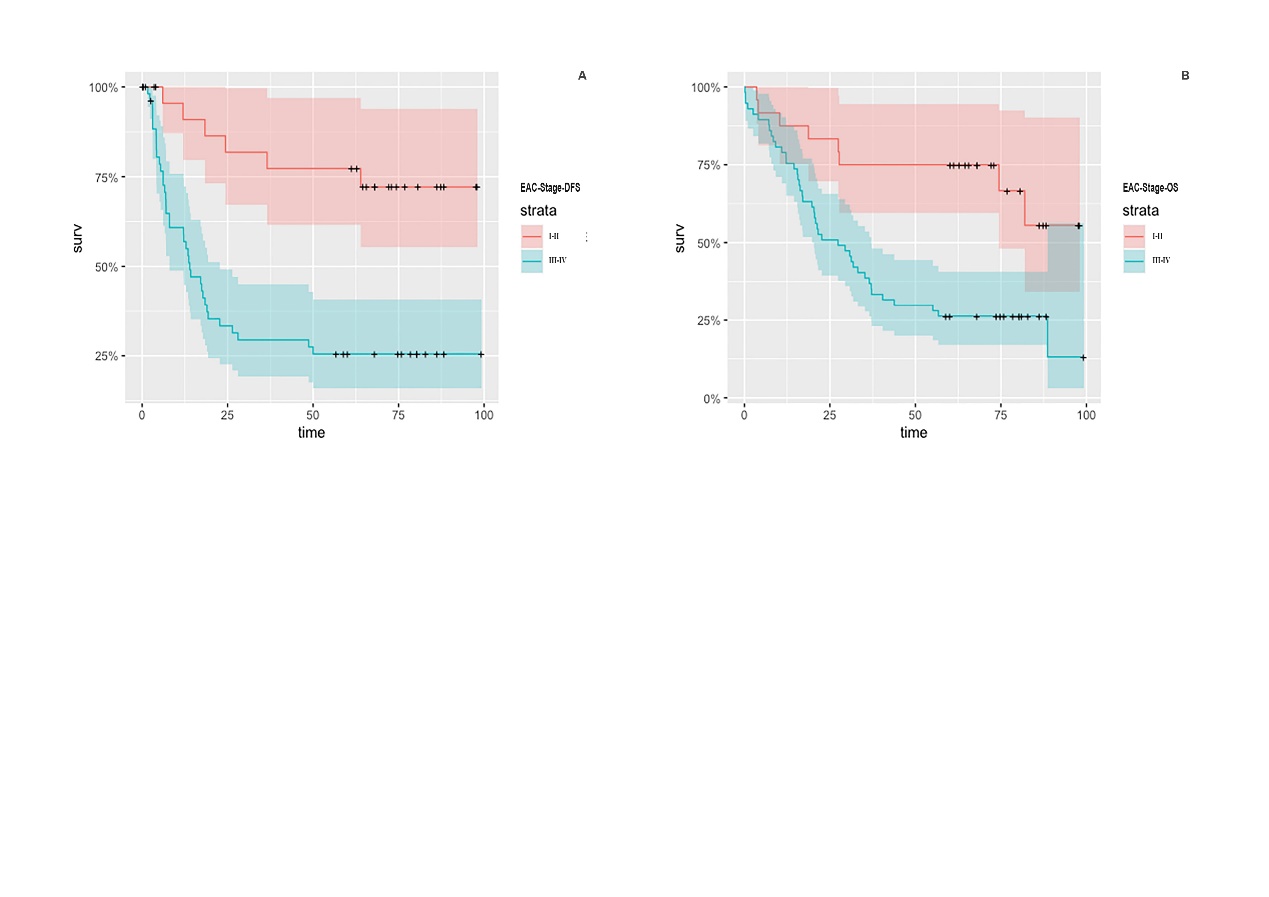


Note: Kaplan Meier log-rank p-value for EAC OS: 0.001 and p-value for EAC DFS< 0.001

Table 4- Overall survival (OS) and Disease- Free survival (DFS)- Multivariate Analysis for the Esophageal AdenoCarcinoma (EAC) EsophagoGastric Junction (EGJ) Siewert I/II subpopulation (N=61/95 patients) including the four SNPs of interest- Bold values denote statistically signiﬁcant associations.

| OS Multivariate Analysis | HR | LCI | HCI | P value |
| --- | --- | --- | --- | --- |
| Age | 1.002 | 0.966 | 1.039 | 0.922 |
| Stage III-IV | 2.815 | 0.980 | 8.089 | 0.055 |
| Neoadjuvant Chemotherapy | 0.606 | 0.245 | 1.501 | 0.279 |
| Adjuvant Chemotherapy | 0.637 | 0.205 | 1.974 | 0.434 |
| Radiotherapy | 0.966 | 0.349 | 2.675 | 0.948 |
| Resection Status: R1 | 0.850 | 0.155 | 4.648 | 0.851 |
| CRM: Positive | 2.262 | 0.727 | 7.037 | 0.159 |
| HOTAIR CT | 1.431 | 0.683 | 2.997 | 0.342 |
| HOTAIR TT | 1.210 | 0.383 | 3.820 | 0.746 |
| LINC00951 AG | 0.743 | 0.347 | 1.588 | 0.443 |
| LINC00951 GG | 0.388 | 0.065 | 2.324 | 0.300 |
| POLR2E CT | 0.585 | 0.235 | 1.457 | 0.249 |
| POLR2E CC | 0.517 | 0.042 | 6.291 | 0.605 |
| HULC AC | 1.311 | 0.533 | 3.226 | 0.556 |
| HULC CC | 1.026 | 0.356 | 2.960 | 0.962 |

| DFS Multivariate Analysis | OR | LCI | HCI | P value |
| --- | --- | --- | --- | --- |
| Age | 0.983 | 0.947 | 1.020 | 0.363 |
| Stage III-IV | 5.423 | 1.376 | 21.375 | **0.016** |
| Neoadjuvant Chemotherapy | 1.156 | 0.447 | 2.988 | 0.764 |
| Adjuvant Chemotherapy | 1.746 | 0.492 | 6.202 | 0.389 |
| Radiotherapy | 1.037 | 0.309 | 3.475 | 0.953 |
| Resection Status: R1 | 0.753 | 0.125 | 4.538 | 0.757 |
| CRM: Positive | 1.264 | 0.366 | 4.371 | 0.711 |
| HOTAIR CT | 1.175 | 0.505 | 2.736 | 0.709 |
| HOTAIR TT | 0.838 | 0.167 | 4.193 | 0.829 |
| LINC00951 AG | 0.793 | 0.324 | 1.939 | 0.611 |
| LINC00951 GG | 0.484 | 0.080 | 2.912 | 0.428 |
| POLR2E CT | 0.733 | 0.278 | 1.933 | 0.530 |
| POLR2E CC | 1.453 | 0.120 | 17.543 | 0.769 |
| HULC AC | 1.402 | 0.473 | 4.153 | 0.542 |
| HULC CC | 1.350 | 0.404 | 4.514 | 0.626 |

Notes: LCI: Lower Confidence Interval, HCI: Higher Confidence Interval, HR: Hazard Ratio, Stage: III-IV versus I-II, Resection Status: R1 versus R0, Circumferential Resection Margin (CRM).

Table 5: Overall survival (OS) and Disease- Free survival (DFS)- Multivariate Analysis for the Esophageal AdenoCarcinoma (EAC) EsophagoGastric Junction (EGJ) Siewert III subpopulation (N=21/95 patients) including the four SNPs of interest (reduced model due to small sample-size)- Bold values denote statistically signiﬁcant associations.

| OS Multivariate Analysis | HR | LCI | HCI | P value |
| --- | --- | --- | --- | --- |
| Stage III-IV | 6.253 | 0.587 | 66.588 | 0.129 |
| Resection Status: R1 | 6.684 | 0.657 | 67.996 | 0.108 |
| HOTAIR CT | 0.982 | 0.168 | 5.732 | 0.983 |
| LINC00951 AG | 1.318 | 0.204 | 8.502 | 0.772 |
| LINC00951 GG | 0.918 | 0.060 | 13.936 | 0.951 |
| POLR2E CT | 1.841 | 0.312 | 10.853 | 0.500 |
| POLR2E CC | 6.663 | 0.334 | 133.010 | 0.214 |
| HULC AC | 0.386 | 0.080 | 1.862 | 0.236 |
| HULC CC | 0.359 | 0.060 | 2.155 | 0.262 |

| DFS Multivariate Analysis | HR | LCI | HCI | P value |
| --- | --- | --- | --- | --- |
| Stage III-IV | 8.067 | 0.939 | 69.294 | 0.057 |
| Resection Status: R1 | 7.360 | 0.734 | 73.766 | 0.090 |
| HOTAIR CT | 0.831 | 0.129 | 5.367 | 0.846 |
| LINC00951 AG | 0.913 | 0.165 | 5.050 | 0.917 |
| LINC00951 GG | 0.355 | 0.033 | 3.861 | 0.395 |
| POLR2E CT | 3.067 | 0.600 | 15.660 | 0.178 |
| POLR2E CC | 2.736 | 0.143 | 52.328 | 0.504 |
| HULC AC | 0.505 | 0.084 | 3.034 | 0.455 |
| HULC CC | 0.372 | 0.060 | 2.310 | 0.289 |

Notes: LCI: Lower Confidence Interval, HCI: Higher Confidence Interval, HR: Hazard Ratio, Stage: III-IV versus I-II, Resection Status: R1 versus R0.
